# The roles of psychological needs satisfaction and impulsivity to parent-child conflict and non-suicidal self-injury

**DOI:** 10.3389/fpsyt.2024.1501983

**Published:** 2024-12-23

**Authors:** Chang Wei, Bao Liu, Yu Wang, Yaping Wang, Qian Xu

**Affiliations:** ^1^ School of Arts and Sciences, Guangzhou Maritime University, Guangzhou, China; ^2^ Student Affairs Department, North China Institute of Aerospace Engineering, Langfang, China; ^3^ School of Education, Hubei University of Science and Technology, Xianning, China; ^4^ College of Preschool Education, Guangzhou Preschool Teachers College, Guangzhou, China

**Keywords:** parent-child conflict, psychological needs satisfaction, impulsivity, non-suicidal self-injury, moderated mediation model

## Abstract

**Introduction:**

Non-suicidal self-injury is a serious health problem among adolescents. However, the association between parent–child conflict and adolescent non-suicidal self-injury and its underlying mechanisms have not been studied sufficiently. Based on the interpersonal model of non-suicidal self-injury, we tested the relationship between parent–child conflict and adolescent non-suicidal self-injury. Furthermore, based on self-determination theory and the diathesis-stress model, we examined whether psychological needs satisfaction mediated the link between parent–child conflict and adolescent non-suicidal self-injury, and if impulsivity moderated this mediating effect.

**Methods:**

Using cross-sectional design, we recruited 656 adolescents (Mage = 13.43; 47% female) from two junior high schools in the Hubei province of China.

**Results:**

The results indicated a positive association between parent–child conflict and adolescent non-suicidal self-injury. Psychological needs satisfaction mediated the relationship between parent–child conflict and adolescent non-suicidal self-injury. High impulsivity strengthened the indirect effect of parent–child conflict on adolescent non-suicidal self-injury. Specifically, high impulsivity strengthened the direct relationship between psychological needs satisfaction and adolescent non-suicidal self-injury and further strengthened the indirect association between parent–child conflict and adolescent non-suicidal self-injury.

**Conclusions:**

Our findings highlight the potential mechanisms underlining the relationship between parent–child conflict and adolescent non-suicidal self-injury. Our findings can inspire educational practitioners to focus on the interaction of family risk factors and individual risk factors when developing intervention programs for adolescent non-suicidal self-injury.

## 1 Introduction

Non-suicidal self-injury refers to the deliberate and direct destruction of one**’**s own body tissue without suicidal intention (1). Previous studies have reported that the estimated prevalence of non-suicidal self-injury among Chinese adolescents ranges from 15.0% to 41.49% (2–4). In a recent study, Wang et al. (3) reported that the prevalence of non-suicidal self-injury in the past 12 months was 27% in a sample of 22,868 Chinese adolescents.

Previous studies have shown that non-suicidal self-injury is associated with adolescents**’** mental health (5, 6). For example, in a longitudinal study of 859 Chinese adolescents, Huang et al. (5) found that non-suicidal self-injury was significantly positively associated with depression 6 and 12 months later. Moreover, non-suicidal self-injury is also strongly association with suicidal behavior (7, 8). Therefore, the risk factors for non-suicidal self-injury urgently need to be explored.

Although researchers have focused on the association between parent-child conflict and adolescent problem behaviors (9, 10), the association of parent-child conflict with adolescent non-suicidal self-injury and the underlying mechanisms of this association have not been sufficiently studied. Thus, the current research aimed to test the relationship between parent-child conflict and adolescent non-suicidal self-injury, as well as the mediating role of psychological need satisfaction and the moderating role of impulsivity in this link. Potential findings of this study may provide insights into the development of effective prevention and intervention programs to reduce the incidence of non-suicidal self-injury in adolescents.

## 2 Literature review

### 2.1 Parent–child conflict and non-suicidal self-injury

Parent–child conflict refers to an aspect of the parent–child relationship characterized by acrimonious or discordant interactions during which both the child and parent display negative behaviors and affect (11). The interpersonal model of non-suicidal self-injury (12) proposes that non-suicidal self-injury can reduce the stress or tension caused by negative interpersonal events. According to this theory, adolescents experiencing parent–child conflict may resort to non-suicidal self-injury to reduce stress or tension.

Empirical evidence also supports this view. Researchers have regularly linked parent–child conflict to various negative emotions associated with non-suicidal self-injury (6, 13–15), such as depression (16–18). Moreover, extensive research has demonstrated the association between negative parent–child relationships and adolescent non-suicidal self-injury (19–22). For example, in a longitudinal study of 2127 U.S. adolescents, Victor et al. (21) found that parental harsh punishment positively predicted non-suicidal self-injury one year later. Similarly, in a study of 673 Chinese adolescents, Deng et al. (20) found a positive association between parent–child conflict and non-suicidal self-injury. Thus, based on the interpersonal model of non-suicidal self-injury (12) and empirical research, we propose the following hypothesis:

Hypothesis 1: Parent–child conflict is associated with adolescent non-suicidal self-injury.

### 2.2 Psychological needs satisfaction as a potential mediator

The self-determination theory (23) postulates that a favorable social environment helps to fulfill individual’s psychological needs, but an unfavorable social environment will inhibit these psychological needs from being met. When psychological needs are not met, individual might develop problem behaviors. According to this theory, parent–child conflict may lead to adolescents’ psychological needs frustration, which could then increase non-suicidal self-injury. Previous studies have confirmed that parent–child conflict is a key risk factor for psychological needs frustration in adolescents (24, 25). Moreover, several studies have shown a link between psychological needs frustration and non-suicidal self-injury (26–29). For example, in a study of 193 Canadian adolescents, Emery et al. (27) found a positive association between low psychological needs satisfaction and non-suicidal self-injury. Similarly, in a study of 1007 Chinese adolescents, Geng et al. (28) found a positive association between unmet psychological needs and non-suicidal self-injury.

Prior studies indicate that psychological needs satisfaction may be a mediator in the relationship between parent–child relationships and problem behaviors in adolescents (24, 30). In a sample of 707 Chinese adolescents, Wang (24) found that psychological needs satisfaction mediated the relationship between parent–child conflict and problem behaviors. Similarly, in a sample of 1767 Chinese adolescents, Sun et al. (30) found that psychological needs satisfaction mediated the link between parent–child relationships and mobile phone addiction tendencies. Thus, based on self-determination theory (23) and empirical research, we propose the following hypothesis:

Hypothesis 2: Psychological needs satisfaction mediates the association between parent–child conflict and adolescent non-suicidal self-injury.

### 2.3 Impulsivity as a moderator

Not all adolescents who experience parent–child conflict engage in non-suicidal self-injury. Therefore, individual factors should also be considered when examining the association between parent–child conflict and non-suicidal self-injury. The diathesis–stress model (31) proposes that individuals with a personality vulnerability may exhibit maladaptive behaviors when exposed to unfavorable environments. Empirical evidence also supports this view. For example, based on this model, Qu et al. (32) found that that punishment sensitivity amplified the negative effect of parental psychological control on non-suicidal self-injury in a sample of 583 adolescents. Similarly, Yu et al. (33) found that cyberbullying victimization were associated with non-suicidal self-injury under the condition of higher sensation seeking in a sample of 1102 adolescents.

Previous studies have shown that impulsivity is an important risk factor for non-suicidal self-injury (34–37). For example, in a study of 267 Italian adolescents, Di Pierro et al. (35) found a positive association between impulsivity and the lifetime presence of non-suicidal self-injury. Similarly, in a longitudinal study of 1686 young people in the UK, Cassels et al. (34) found that impulsivity positively predicted non-suicidal self-injury one year later. According to the diathesis–stress model (31), with high impulsivity may exhibit non-suicidal self-injury when exposed to parent–child conflict. Indeed, high impulsivity has been shown to amplify the risk effect of unfavorable environments on adolescent non-suicidal self-injury (38, 39). For example, in a sample of 1870 adolescents, Ye et al. (39) found that that high impulsivity amplified the negative effect of bullying on non-suicidal self-injury. Thus, based on the diathesis–stress model (31) and empirical research, we propose the following hypothesis:

Hypothesis 3: Impulsivity strengthens the indirect relationship between parent–child conflict and adolescent non-suicidal self-injury through psychological needs satisfaction.

### 2.4 The present study

**Figure 1:**
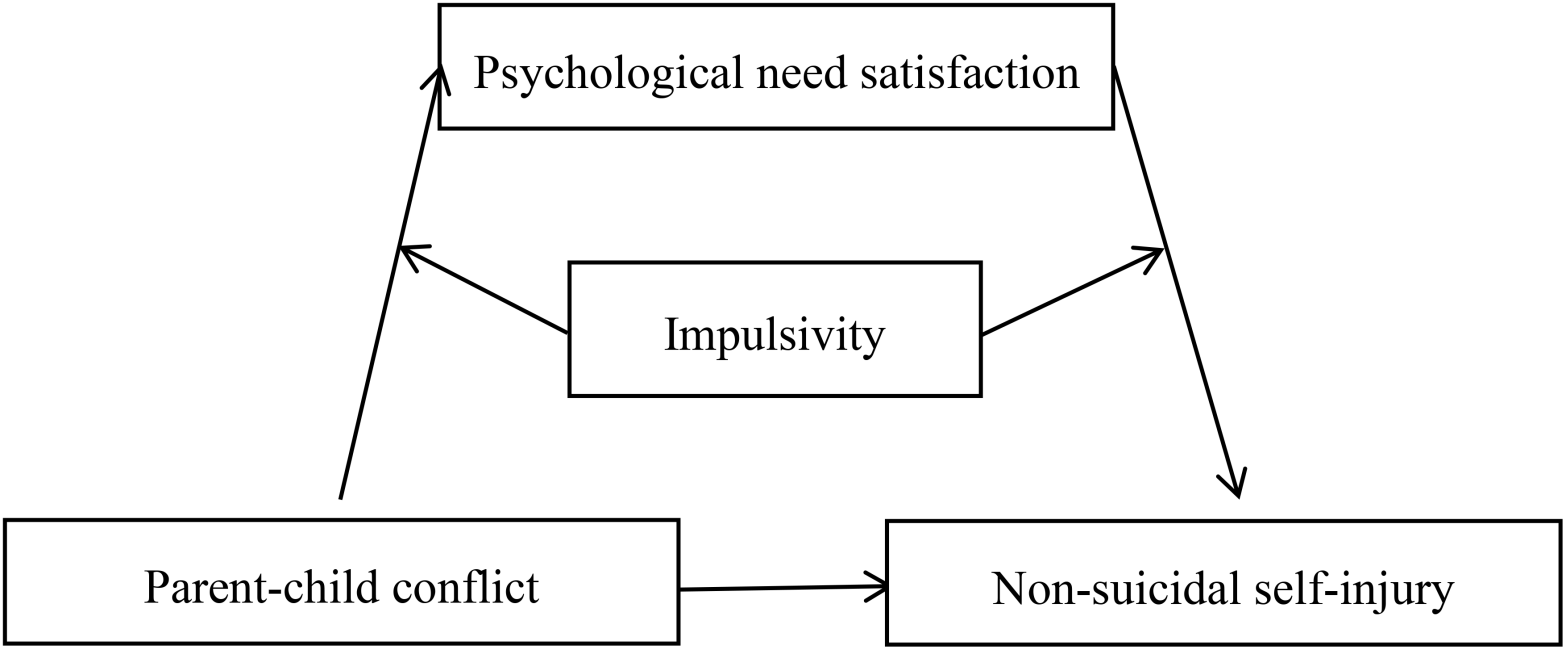
The proposed moderated mediation model.

Using a cross-sectional design, grounded in the interpersonal model of non-suicidal self-injury (12), the current study examined the association between parent–child conflict and adolescent non-suicidal self-injury (Hypothesis 1). Furthermore, based on self-determination theory (23) and the diathesis–stress model (31), this study tested whether psychological needs satisfaction mediates the link between parent–child conflict and adolescent non-suicidal self-injury (Hypothesis 2), and whether this mediating process is moderated by impulsivity (Hypothesis 3). **Figure 1** presents the proposed model.

## 3 Method

### 3.1 Participants and procedure

Using a cross-sectional design, we recruited participants from two junior high schools in Hubei Province, China. The sample included 656 adolescents (*M_age_* = 13.43; *SD* = 0.75), of whom 348 were boys (53%) and 308 were girls (47%). Among this sample, 23.5% of the participants were from rural areas, 61.5% from township areas, and 15.0% from urban areas. In addition, 80.5% of participants’ mothers and 74.9% of their fathers had not completed a high school education.

This study was approved by the Academic Ethics Review Board of school of Arts and Sciences, Guangzhou Maritime University. We obtained informed consent from adolescents, parents, and teachers before data collection. Well-trained psychology teachers collected the data. Students who do not agree to participate in the questionnaire or who are not in school due to leave are excluded. We explained to the participants that they could withdraw from the study at any time during data collection without penalty. They could skip any items they feel uncomfortable with, and their responses would be kept confidential. Some questionnaire items may trigger negative emotions, and psychologists can provide timely psychological counseling. All participants completed the measures in their classrooms and received a signature pen.

### 3.2 Measures

#### 3.2.1 Parent–child conflict

Parent–child conflict was assessed using the Conflict Frequency Subscale of the Parent-Child Conflict Scale (40). Previous studies have shown that the reliability of the scale is good (20). Participants were asked to report the frequency conflicts had occurred between themselves and their parents in the past six months. This scale includes eight items (e.g., ***“*** Have you ever had any conflicts with your parents about how to spend money? ***”***) rated on a 5-point scale (1 = ***“*** none ***”*** to 5 = ***“*** several times a day ***”***). Mean scores were used for analysis, with higher scores indicating higher levels of parent–child conflict. In this study, Cronbach ***’*** s alpha was.84.

#### 3.2.2 Psychological needs satisfaction

Psychological needs satisfaction was assessed using the Psychological Needs Satisfaction Scale (41). Previous studies have shown that the reliability of the scale is good (28). This scale includes 21 items (e.g., ***“*** I feel free to express my thoughts and opinions ***”***) scored on a 5-point scale (1 = ***“*** not at all true ***”*** to 5 = ***“*** very true ***”***). Mean scores were used for analysis, with higher scores indicating higher levels of psychological needs satisfaction. In this study, Cronbach ***’*** s alpha was.82.

#### 3.2.3 Impulsivity

Impulsivity was assessed using the Impulsivity Subscale of the Chinese Version of the Dual-Mode of Self-Control Scale (42), which was adapted from the Dual-Mode of Self-Control Scale (43). Previous studies have shown that the reliability of the scale is good (44). This scale includes six items (e.g., ***“*** I ***’*** m an impulsive person ***”***) rated on a 5-point scale (1 = ***“*** not at all true ***”*** to 5 = ***“*** very true ***”***). Mean scores were used for analysis, with higher scores indicating a higher impulsivity. In this study, Cronbach ***’*** s alpha was.85.

#### 3.2.4 Non-suicidal self-injury

Non-suicidal self-injury was assessed using the Non-Suicidal Self-Injury Scale (45), which was adapted from the Deliberate Self-Harm Inventory (46). Previous studies have shown that the reliability of the scale is good (47). This scale includes 12 items (e.g., ***“*** cutting oneself, carving oneself, and burning oneself ***”***) rated on a 6-point scale (0 = ***“*** never ***”*** to 5 *=* ***“*** 5 or more times ***”***). Mean scores were used for analysis, with higher scores indicating more frequent non-suicidal self-injury. In this study, Cronbach ***’*** s alpha was.84.

### 3.3 Statistical analyses

Prior research indicates that age and gender are correlated with non-suicidal self-injury (48, 49). Therefore, we controlled for age and gender in analyses. Missing data were handled with mean imputation. This method uses the mean of the score to fill in the missing data. We used SPSS 21.0 to examine descriptive statistics and correlations. We used the PROCESS macro (Model 4) developed by Hayes (50) in SPSS to examine whether psychological needs satisfaction mediated the association between parent–child conflict and non-suicidal self-injury. We also used the PROCESS macro (Model 58) to analyze the moderating role of impulsivity in the indirect relationship between parent–child conflict and non-suicidal self-injury via psychological needs satisfaction.

## 4 Results

### 4.1 Preliminary analyses

**Table 1** presents the means, standard deviations, and correlation coefficients for all study variables. Parent–child conflict (*r* **= .**14, *p* <.001) and impulsivity (*r* **= .**21, *p* <.001) were both significantly positively correlated with non-suicidal self-injury. Parent–child conflict was significantly negatively related to psychological needs satisfaction (*r* = -.14, *p* <.001), while psychological needs satisfaction was significantly negatively related to non-suicidal self-injury (*r* = -.18, *p* <.001).

**Table 1:** Descriptive statistics and correlations for all variables.

Variable	1	2	3	4	5	6
1. Gender	1.00					
2. Age	0.13***	1.00				
3. Parent-child conflict	-0.04	0.03	1.00			
4. Psychological needs satisfaction	0.07	0.02	-0.14***	1.00		
5. Impulsivity	-0.16***	-0.03	0.18***	-0.27***		
6. Non-suicidal self-injury	-0.14***	0.03	0.14***	-0.18***	0.21***	1.00
*Mean*	0.53	13.43	1.91	3.25	2.40	1.56
*SD*	0.50	0.75	0.76	0.48	0.79	0.42

Gender was dummy coded as 1 = male, 0 = female. ***p <.001.

### 4.2 Mediating effect of psychological needs satisfaction

**Figure 2:**
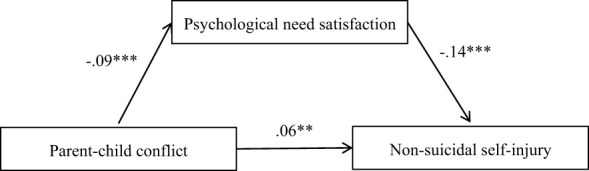
Model of the mediating role of psychological needs satisfaction in the association between parent-child conflict and non-suicidal self-injury. **p <.01. ***p <.001.

**Figure 2** displays the results for each path in the proposed model. After controlling for age and gender, parent–child conflict was found to positively predict non-suicidal self-injury (*b* = .06, *p* <.01) and negatively predict psychological needs satisfaction (*b* = -.09, *p* <.001). Furthermore, psychological needs satisfaction negatively predicted non-suicidal self-injury (*b* = -.14, *p* <.001). The indirect effect of parent–child conflict on non-suicidal self-injury through psychological needs satisfaction was.012, 95% CI = [.004,.023]. Details are shown in **Table 2**.

**Table 2:** The mediation effect of parent-child conflict on non-suicidal self-injury.

	Model 1 (PNS)				Model 2 (NSSI)			
	*b*	*SE*	*t*	95% CI	*b*	*SE*	*t*	95% CI
Gender	0.06	0.04	1.68	[−0.01, 0.14]	-0.11	0.03	-3.32***	[−0.17, -0.04]
Age	0.01	0.02	0.36	[-0.04, 0.06]	0.02	0.02	1.17	[−0.02, 0.07]
PCC	-0.09	0.02	-3.68***	[-0.14, -0.04]	0.06	0.02	2.99**	[0.02, 0.10]
PNS					-0.14	0.03	-4.04***	[-0.20, -0.07]
R^2^	0.03				0.06			
F	5.71***				10.97***			

Gender was dummy coded as 1 = male, 0 = female. PCC = parent-child conflict; PNS = psychological needs satisfaction; NSSI = non-suicidal self-injury. **p <.01. ***p <.001.

### 4.3 Moderated mediation analysis

**Figure 3:**
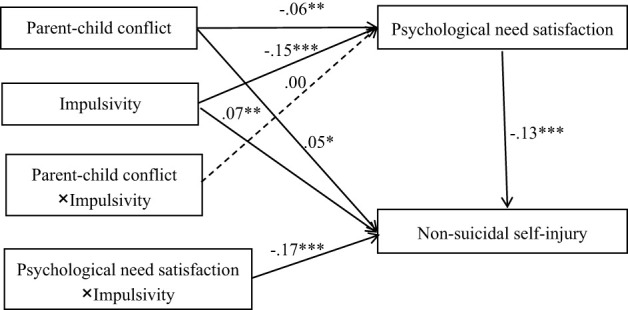
Model of the moderating role of impulsivity on the indirect relationship between parent-child conflict and non-suicidal self-injury. *p <.05. **p <.01. ***p <.001.

**Figure 3** displays the results for each path in the proposed model. After controlling for age and gender, parent–child conflict predicted psychological needs satisfaction (*b* = -.06, *p* <.01) and non-suicidal self-injury (*b* **= .**05, *p* <.05). Psychological needs satisfaction (*b* = -.13, *p* <.001) and impulsivity (*b* **= .**07, *p* <.01) predicted non-suicidal self-injury. Moreover, impulsivity significantly moderated the effects of psychological needs satisfaction on non-suicidal self-injury (*b* = -.17, *p* <.001). Details are shown in **Table 3**.

**Table 3:** The moderated mediation effect of parent-child conflict on non-suicidal self-injury.

	Model 1 (PNS)				Model 2 (NSSI)			
	*b*	*SE*	*t*	95% CI	*b*	*SE*	*t*	95% CI
Gender	0.02	0.04	0.56	[−0.05, 0.09]	-0.08	0.03	-2.63**	[−0.15, -0.02]
Age	0.01	0.02	0.41	[-0.04, 0.06]	0.02	0.02	1.04	[−0.02, 0.06]
PCC	-0.06	0.02	-2.61**	[-0.11, -0.02]	0.05	0.02	2.44*	[0.01, 0.09]
Impulsivity	−0.15	0.02	−6.33***	[−0.20, −0.10]	0.07	0.02	3.15**	[0.02, 0.11]
PCC × Impulsivity	0.00	0.03	0.04	[−0.05, 0.06]				
PNS					-0.13	0.03	-3.84***	[-0.20, -0.06]
PNS × Impulsivity					-0.17	0.03	-4.92***	[−0.24, -0.10]
R^2^	0.08				0.12			
F	11.51***				13.89***			

Gender was dummy coded as 1 = male, 0 = female. PCC = parent-child conflict; PNS = psychological needs satisfaction; NSSI = non-suicidal self-injury. *p < 0.05. **p <.01. ***p <.001.

**Figure 4:**
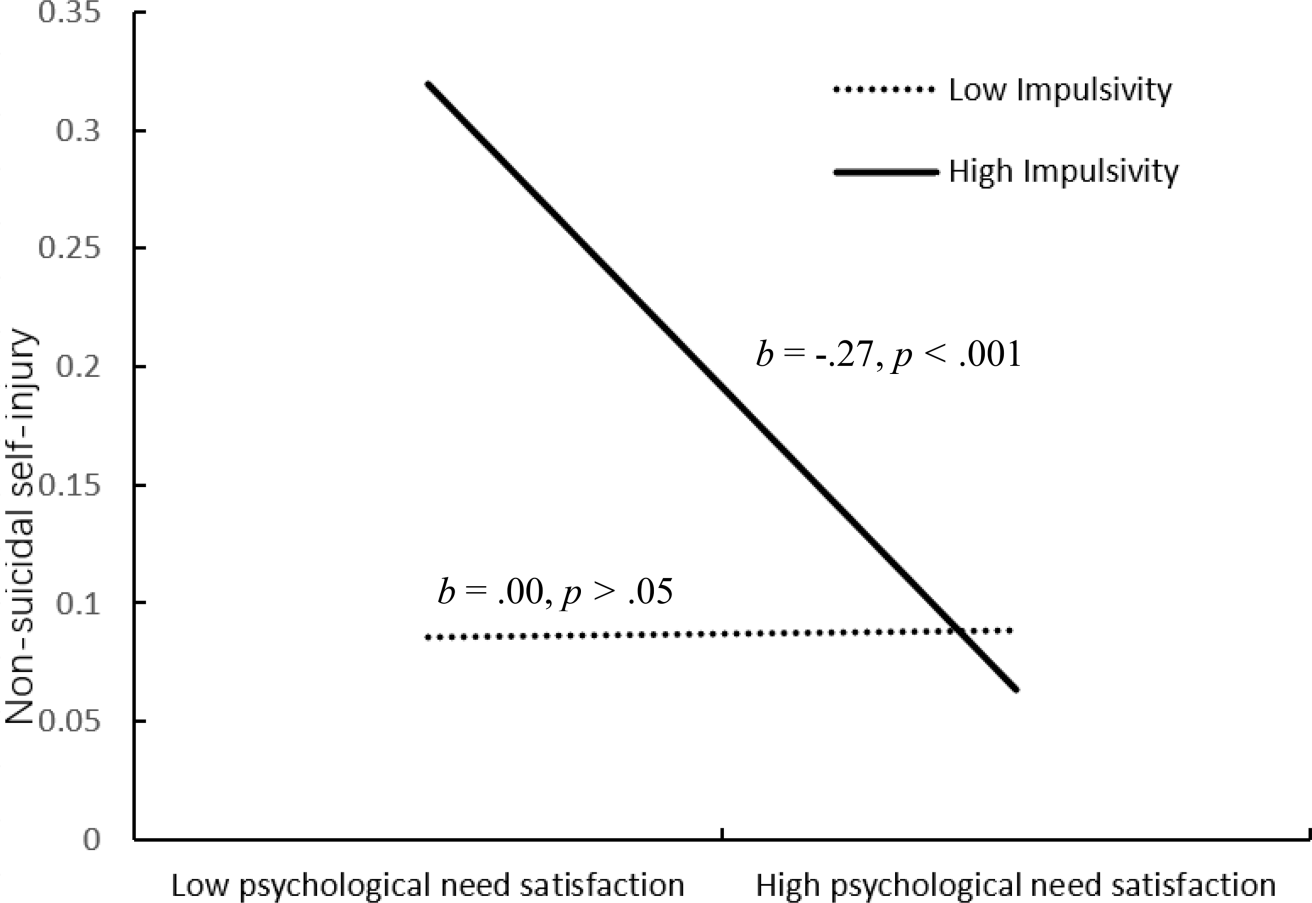
Interactive effect of psychological need satisfaction and impulsivity on non-suicidal self-injury.

We conducted simple slopes tests to better understand the results with impulsivity as a moderator. As shown in **Figure 4**, the association between psychological needs satisfaction and non-suicidal self-injury was significant when participants reported higher impulsivity (*b* = -.27, *p* <.001), but not significant when they reported lower impulsivity (*b* **= .**00, *p* >.05).

## 5 Discussion

Based on the interpersonal model of non-suicidal self-injury (12), self-determination theory (23) and the diathesis–stress model (31), the current study examined the association between parent–child conflict and adolescent non-suicidal self-injury, as well as the mediating role of psychological needs satisfaction and the moderating role of impulsivity. As expected, we found that parent–child conflict was positively associated with non-suicidal self-injury. Furthermore, psychological needs satisfaction mediated this association, and high impulsivity strengthened the indirect effect.

### 5.1 Parent–child conflict and non-suicidal self-injury

Consistent with Hypothesis 1, we found that parent–child conflict was significantly positively correlated with adolescent non-suicidal self-injury. Thus, adolescents who experience parent–child conflict are more likely to engage in non-suicidal self-injury. This finding shows that parent–child conflict is a risk factor for adolescent non-suicidal self-injury. Our results are consistent with those reported in previous studies indicating that negative parent–child relationships could increase the risk of adolescent non-suicidal self-injury (19, 20, 22, 51, 52). Furthermore, our findings are congruent with the interpersonal model of non-suicidal self-injury (12). According to this theory, parent–child conflict, as a negative interpersonal event, can lead to stress or tension, which in turn increases the risk of adolescent non-suicidal self-injury (1, 12). This study used non-suicidal self-injury as an indicator of adolescent maladjustment and found that individuals engage in non-suicidal self-injury to cope with parent–child conflict. These findings suggest that reducing parent–child conflict would be beneficial in reducing adolescent non-suicidal self-injury.

### 5.2 Mediating role of psychological needs satisfaction

Consistent with Hypothesis 2 and self-determination theory (23), we found that psychological needs satisfaction mediated the relationship between parent–child conflict and adolescent non-suicidal self-injury. This indicates that psychological needs satisfaction is a key bridge linking parent–child conflict to non-suicidal self-injury. Therefore, parent–child conflict may lead to adolescents’ psychological needs frustration, which could then increase problem behaviors such as non-suicidal self-injury. Earlier studies have demonstrated that psychological needs satisfaction is independently associated with both parent–child conflict and non-suicidal self-injury (24, 28, 29, 53). The current study used a cross-sectional design to demonstrate the indirect role of psychological needs satisfaction in the association between parent–child conflict and adolescent non-suicidal self-injury, extending past research on the mediating role of psychological needs satisfaction in the association between cyberbullying victimization and adolescent non-suicidal self-injury (28), and between parent–child conflict and other forms of problem behaviors (24). This finding expands the findings of previous studies that tested this association (20, 52) and highlights the important role of psychological needs satisfaction in understanding problem behaviors in adolescents, such as non-suicidal self-injury.

### 5.3 Moderating role of impulsivity

Consistent with Hypothesis 3, we found that impulsivity strengthened the relationship between psychological needs satisfaction and non-suicidal self-injury and increased the indirect association between parent–child conflict and non-suicidal self-injury. Specifically, high impulsivity strengthened the connection between low psychological needs satisfaction and non-suicidal self-injury. A potential explanation for these findings is that parent–child conflict may lead to adolescents’ psychological needs frustration (24, 25), which in turn could lead to negative emotional states such as anxiety and depression (54–56). Adolescents with high impulsivity have poor emotional management skills (39) and are more likely to use non-suicidal self-injury as a maladaptive coping strategy to alleviate symptoms of anxiety and depression (1, 6, 15). These results align with the diathesis–stress model (31) and provide further evidence that high impulsivity magnifies the indirect mechanism through which parent–child conflict leads to adolescent non-suicidal self-injury. Our results are consistent with those reported in previous studies indicating that impulsivity could amplify the negative effect of adverse environments on adolescent non-suicidal self-injury (38, 39).

### 5.4 Implications for practice

The results of this study have several important implications. First, given that parent–child conflict is a risk factor for adolescent non-suicidal self-injury, parents need to increase their awareness and prevention efforts to reduce conflict with their children. Family cooperation strategies can be used to alleviate parent–child conflict through active mediation by a third party (57). For example, when there is a conflict between father and son, the mother intervenes in time to interrupt the conflict, so that the conflict tends to de-escalate. In addition, adolescents and parents can participate in family therapy programs (SHIFT), which can effectively enhance friendly communication within the family, thereby reducing parent-child conflict. (58). Second, this study found that psychological needs satisfaction mediates the association between parent-child conflict and adolescent non-suicidal self-injury, emphasizing the importance of psychological needs satisfaction in interventions for adolescent non-suicidal self-injury. Schools can help meet adolescents’ psychological needs by improving the quality of teacher–student relationships and peer relationships (59). For example, the quality of teacher-student relationships and peer relationships can be improved by promoting interaction between class members through themed class meetings (60). Third, the results showed that impulsivity is a risk factor that amplifies the indirect relationship between parent–child conflict and adolescent non-suicidal self-injury through psychological needs satisfaction. Therefore, practitioners can reduce adolescents’ impulsivity by improving their emotional management and impulse control skills, thereby helping to reduce adolescent non-suicidal self-injury (39). For example, psychotherapists can employ group cognitive therapy methods to improve emotional management and reduce impulsivity (61).

### 5.5 Limitations and future directions

This study had several limitations. First, the cross-sectional design cannot establish the temporal relationships between studied variables, therefore, inferred causality cannot be tested. Although the conceptual model was based on strong theoretical frameworks and empirical evidence, the possibility of bidirectional relationships between certain research variables cannot be ruled out. For example, previous studies have shown the relationship between parent-child relationships and adolescent problem behaviors is bidirectional (62). Thus, future studies should use a longitudinal design to explore the bidirectional relationships between parent-child conflict, psychological needs satisfaction, impulsivity and non-suicidal self-injury. Second, this study used self-report data from adolescents’, which provided subjective information, and the results could be influenced by common method bias and social desirability. Future research should use multiple information sources (e.g., parents’ report, peers’ report and teachers’ report) for data collection. Third, this study only examined the mediating role of psychological needs satisfaction in the relationship between parent–child conflict and non-suicidal self-injury. Future research could examine the mediating roles of other variables, such as self-esteem (39, 63), self-compassion (64), and emotional insecurity (2). Fourth, we examined the moderating effect of impulsivity on the relationship between parent–child conflict and non-suicidal self-injury; however, we did not examine the effects of other individual variables. Future research should examine the effects of multiple individual variables, such as self-control (4), gratitude (65, 66), sensation-seeking (33, 67) and physical development (68, 69). Fifth, the sample used in this study was recruited from China; therefore, future studies should recruit samples from different countries to test the generalizability of the findings.

## 6 Conclusions

Current study highlights the roles of psychological needs satisfaction and impulsivity in the association between parent–child conflict and adolescent non-suicidal self-injury. psychological needs frustration served as a mechanism by which parent–child conflict was associated with adolescent non-suicidal self-injury. Moreover, the risk effect of impulsivity was manifested in the second stage of the indirect relationship between parent–child conflict and adolescent non-suicidal self-injury.

## Data Availability

The original contributions presented in the study are included in the article/supplementary material. Further inquiries can be directed to the corresponding authors.
